# Carbon, nitrogen, and phosphorus storage in alpine grassland ecosystems of Tibet: effects of grazing exclusion

**DOI:** 10.1002/ece3.1732

**Published:** 2015-09-23

**Authors:** Xuyang Lu, Yan Yan, Jian Sun, Xiaoke Zhang, Youchao Chen, Xiaodan Wang, Genwei Cheng

**Affiliations:** ^1^ Key Laboratory of Mountain Surface Processes and Ecological RegulationChinese Academy of SciencesChengdu610041China; ^2^ Xainza Alpine Steppe and Wetland Ecosystem Observation and Experiment StationChinese Academy of SciencesXainza853100China; ^3^ Key Laboratory of Ecosystem Network Observation and ModelingChinese Academy of SciencesBeijing100101China

**Keywords:** Alpine meadow, alpine steppe, fencing, soil organic carbon, Tibetan Plateau

## Abstract

In recent decades, alpine grasslands have been seriously degraded on the Tibetan Plateau and grazing exclusion by fencing has been widely adopted to restore degraded grasslands since 2004. To elucidate how alpine grasslands carbon (C), nitrogen (N), and phosphorus (P) storage responds to this management strategy, three types of alpine grassland in nine counties in Tibet were selected to investigate C, N, and P storage in the environment by comparing free grazing (FG) and grazing exclusion (GE) treatments, which had run for 6–8 years. The results revealed that there were no significant differences in total ecosystem C, N, and P storage, as well as the C, N, and P stored in both total biomass and soil (0–30 cm) fractions between FG and GE grasslands. However, precipitation played a key role in controlling C, N, and P storage and distribution. With grazing exclusion, C and N stored in aboveground biomass significantly increased by 5.7 g m^−2^ and 0.1 g m^−2^, respectively, whereas the C and P stored in the soil surface layer (0–15 cm) significantly decreased by 862.9 g m^−2^ and 13.6 g m^−2^, respectively. Furthermore, the storage of the aboveground biomass C, N, and P was positively correlated with vegetation cover and negatively correlated with the biodiversity index, including Pielou evenness index, Shannon–Wiener diversity index, and Simpson dominance index. The storage of soil surface layer C, N, and P was positively correlated with soil silt content and negatively correlated with soil sand content. Our results demonstrated that grazing exclusion had no impact on total C, N, and P storage, as well as C, N, and P in both total biomass and soil (0–30 cm) fractions in the alpine grassland ecosystem. However, grazing exclusion could result in increased aboveground biomass C and N pools and decreased soil surface layer (0–15 cm) C and P pools.

## Introduction

Grasslands occupy approximately 37–50% of terrestrial land cover and are estimated to contain more than one‐third of the world's carbon (C) reserves, which play an important role in the global C cycle (Menke and Bradford 1992; Haferkamp and Macneil 2004; Reynolds et al. 2005; Mara 2012). Globally, grasslands have been reported to sequester C in the soil at a rate of 0.5 Pg C year^−1^, which is about one‐fourth of the potential C sequestration in soil worldwide (Wu et al. 2014a). A small percentage change in C storage in grasslands could have a large impact on atmospheric carbon dioxide (CO_2_) and global C cycling and balance. The maintenance of C storage is also a key factor in the sustainability of grassland ecosystems (Wang et al. 2014). Nevertheless, grasslands are one of the most modified biomes on Earth; a large proportion of grasslands have been replaced by crop fields or are subject to livestock grazing (Piñeiro et al. 2006). Grasslands may have high C sequestration potential if the input of organic matter into the soil and the reduction of soil organic matter decomposition are promoted through reasonable management practices, but many grasslands receive low C inputs and tend to be degraded, poorly managed, or not managed at all (Shrestha and Stahl 2008).

Grazing exclusion is an effective grassland management practice that aims to prevent grassland degradation and retain grassland ecosystem function. It has been widely regarded as a method to restore vegetation and soil in degraded grassland ecosystems throughout the world in recent decades (Schultz et al. 2011; Medina‐Roldán et al. 2012). Numerous studies have demonstrated that grazing exclusion can cause measurable changes in biogeochemical properties of the soil (Medina‐Roldán et al. 2012; Mekuria and Aynekulu 2013). However, effects of grazing exclusion on soil C storage and accumulation are controversial. In some cases, grazing exclusion has a positive effect on soil C storage (Raiesi and Riahi 2014; Speed et al. 2014), in some a negative effect (Hafner et al. 2012; Shi et al. 2013), and in some a neutral effect (Shrestha and Stahl 2008; Medina‐Roldán et al. 2012). For instance, grazing exclusion increased soil C pools in a semiarid woody rangeland in the Zagros Mountains of central Iran (Raiesi and Riahi 2014), decreased them in a mountain pasture of *Kobresia* on the northeastern Tibetan Plateau (Hafner et al. 2012), and had no impact in upland grassland in northern England (Medina‐Roldán et al. 2012). Lack of a clear relationship between grazing exclusion and soil C storage may result from differences between grassland ecosystem types, inconsistent duration of grazing exclusion, soil heterogeneity, and different environmental conditions between studies (Mekuria and Aynekulu 2013; Luan et al. 2014; Raiesi and Riahi 2014).

Nitrogen (N) and phosphorus (P) have long been recognized as nutrients that commonly limit or co‐limit plant growth and net primary production throughout the terrestrial biosphere (Dijkstra et al. 2012; Laliberté et al. 2015). Grazing exclusion also has variable effects on soil N and P cycling and storage. For instance, total N storage, including N stored in aboveground biomass, litter, roots, and soil, all increased significantly due to grazing exclusion in a semiarid grassland region in northern China (He et al. 2008; Li et al. 2012). N stored in aboveground biomass and litter significantly increased, but no changes in N stored in belowground biomass or soil N following grazing exclusion were reported in the grassland on the Loess Plateau in China (Wang et al. 2014). Similarly, soil N storage was reported not altered by grazing exclusion in a grassland region in the central Monte Desert in Argentina (Gómez et al. 2012). Whereas in a sandy rangeland in western Oklahoma of America, total N storage in litter was found significantly increased, but total N storage in the surface 5 cm of soil significantly decreased due to grazing exclusion (Berg et al. 1997). For potential ecosystem P storage, Mekuria and Aynekulu (2013) found that soil available P stocks increased 26–39% due to grazing exclusion on grazing lands in the northern highlands of Ethiopia, and Chaneton et al. (1996) found that total P stored in vegetation did not differ between grazed and ungrazed grassland in the Flooding Pampa, Argentina.

The alpine grasslands of the Tibetan Plateau, the most expansive areas of alpine grassland in the world, store 2.5% of the global pool of soil C (Luan et al. 2014). Alpine grasslands have traditionally served as the principal pastures for Tibetan communities and also provide ecosystem functions and services, such as biodiversity conservation and soil and water protection (Wang et al. 2002; Wen et al. 2013a,b). In recent decades, alpine grasslands have been seriously degraded by human activities and climate change (Harris 2010). In response to the problem of grassland degradation in the Tibetan Plateau, China's state and local authorities initiated a program in 2004 called “retire livestock and restore grassland”. This campaign has focused mostly on the use of protective fencing enclosures to exclude livestock from alpine grasslands within the traditional grazing regions. In this study, three types of alpine grassland in nine counties in Tibet were selected as sampling sites to evaluate the effects of grazing exclusion on ecosystem C, N, and P storage. Nine counties, in which the extent of fenced area was relatively large, represented three of the main natural grassland vegetation types in Tibet, including alpine meadow, alpine steppe, and alpine desert steppe. We tested the hypothesis that grazing exclusion is associated with an increase in ecosystem C, N, and P storage. Such increases are characterized by improvements in vegetation biomass C, N, and P pools and soil C, N, and P pools due to absence of disturbance by livestock.

## Materials and Methods

### Study area

Tibet is located between 26°50′ and 36°29′ N and 78°15′ and 99°07′ E and covers a total area of more than 1.2 million km^2^, which is approximately one‐eighth of the total area of China. It is an important ecological shelter zone that acts as an important reservoir for water; this region also regulates climate change and water resources in China and eastern Asia (Immerzeel et al. 2010; Qian et al. 2011). The region has a diverse range of climate zones due to its extensive territory and highly dissected topography. Solar radiation is strong; the annual radiation varies between 140 and 190 kcal cm^−2^. Sunshine hours are long, with annual sunshine ranging from 1800 to 3200 h. The average annual temperature is rather low, with a large diurnal range, and varies from 18°C to −4°C. The average annual precipitation is less than 1000 mm in most areas of Tibet; annual precipitation rates can reach up to 2817 mm in the east and drop down to approximately 70 mm in the west (Dai et al. 2011).

Alpine grasslands are the most dominant ecosystems of Tibet, covering more than 70% of the plateau. According to the first national survey of Chinese grassland resources, the grasslands of the Tibetan Plateau are generally classified into 17 types based on climatic zonation, humidity index, vegetation type, and importance to the livestock industry (Ni 2002; Fan et al. 2008). Alpine steppe is the most common grassland type in Tibet; it is composed of drought‐tolerant perennial herbs or small shrubs under cold and arid and semiarid climate conditions; approximately 38.9% of the total Tibetan grassland area meets this definition. Alpine meadow is the second largest grassland type, and is composed of perennial mesic and ‘mesoxeric herbs’ under cold and wet climate conditions; it occupies approximately 31.3% of the total grassland area of Tibet. Alpine desert steppe occupies approximately 10.7% of the total grassland area, and is composed by xeric small shrubs and small grasses under cold and arid climate conditions; this is a transitional type of alpine grassland that occurs in areas between the steppe and the desert (Land Management Bureau of Tibet, 1994).

### Survey design and sampling

In late July to mid‐August in 2013, we conducted a multisite survey during the peak growing season in nine counties, and in these the extent of the fenced area was relatively larger and represented the following three major natural grassland vegetation types in Tibet: alpine meadow, alpine steppe, and alpine desert steppe (Table 1). To restore degraded alpine grasslands, grazing enclosures by metal fences (mostly larger than 30 hm^2^ in size) were established in 2005–2007 in these counties, supported by the “retire livestock and restore grassland” program (Wu et al. 2014b). Since fencing establishment, the fenced plot has completely been excluded from livestock grazing, while in the surrounding grassland conventional grazing by yak, sheep, and goats around the year has continued. The fencing is also effective to exclude large wildlife herbivores, such as *Pantholops hodgsonii*,* Procapra picticaudata*, and *Equus kiang*, but ineffective to small ones such as pika and marmot (Wu et al. 2014b;). The enclosed areas inside the fences were defined as grazing exclusion (GE) plots. The areas outside of the fences nearby were defined as free grazing (FG) plots.

**Table 1:** Description of the alpine grassland sampling sites in Tibet

Alpine Grassland type	Location	Longitude (E)	Latitude (N)	Altitude (m)	Dominant species	GST (°C)	GSP (mm)
Alpine meadow	Damxung	91°14′56″	30°36′08″	4407	*Kobresia pygmaea* C. B. Clarke	5.9	433.3
	Nagqu	92°09′11″	31°16′30″	4458	*Kobresia humilis*	5.7	480.0
	Nierong	92°16′49″	32°07′48″	4614	*Kobresia littledalei* C. B. Clarke	6.2	449.8
	Ando	91°38′28″	32°15′37″	4696	*Kobresia pygmaea* C. B. Clarke	6.5	425.1
Alpine steppe	Baingoin	92°09′11″	31°16′30″	4632	*Carex moorcroftii* Falc. Ex Boott	6.6	379.3
	Nima	87°24′57″	31°48′27″	4550	*Stipa purpurea*	6.9	337.2
	Coqen	85°09′09″	31°01′58″	4687	*Stipa purpurea*	6.1	245.4
	Ngamring	86°37′52″	29°38′38″	4583	*Carex moorcroftii* Falc. Ex Boott	4.7	305.7
Alpine desert steppe	Gêrzê	84°49′34″	31°59′25″	4591	*Stipa purpurea*	7.5	196.1

GST, growing season temperature; GSP, growing season precipitation.

In order to investigate the effect of grazing exclusion on ecosystem C, N, and P storage in alpine grasslands, three pairs of 0.5 m × 0.5 m quadrats at each of the GE and FG treatment sample plots were laid out along a sample line at intervals of approximately 20 m. The quadrats of GE plots chosen in this study were well matched with the adjacent FG plots, and both quadrats in GE and FG plots are within 800 m from the enclosure edges to make sure that each pair sites were as similar as possible in slope, aspect, and soils. All species within each quadrat were identified, and their coverage, density, frequency, and height were measured. Aboveground and belowground plant components were harvested. Aboveground plant parts within the sample quadrat were clipped to the soil surface with scissors. Belowground plant parts in the sample quadrat were directly acquired by excavation. After plant samples were sun‐dried in the field, they were brought to the laboratory and oven‐dried at 65°C for 72 h to determine biomass and C, N, and P contents. Soil samples were taken from FG plots and GE plots at the following two depths: 0–15 cm and 15–30 cm. For the determination of soil bulk density, soil cores (5.4 cm in diameter) were taken from each layer using a stainless steel cylinder. In addition, the location and elevation of each site were measured using GPS (Garmin MAP62CSX made in Garmin Ltd, Olathe, Kansas, USA).

### Plant and soil sample analysis

Soil bulk density was determined as the moisture‐corrected (oven‐dried at 105°C) mass of each sample divided by the measured volume of the excavated soil core (Campbell et al. 2014). Soil samples for physical and chemical properties measurements were air‐dried, crushed, and passed through a 2‐mm‐mesh sieve. Coarse materials, such as gravel and roots, were removed. Samples from the <2 mm fraction were weighed and used for analyses. Soil particle size distributions were determined by the pipette method, which followed H_2_O_2_ treatment to destroy organic matter and the dispersion of soil suspensions by sodium hexametaphosphate (Su et al. 2010). Soil pH was determined in a soil to deionized water suspension of 1:2.5 (w/v) (Alvarenga et al. 2012). Plant C, N, and soil organic carbon (SOC) and total N contents were determined using a vario MACRO cube elemental analyzer (Elementar Analysensysteme GmbH, German) (Qu et al. 2014). To remove inorganic carbon, all samples for SOC analysis were acid treated with hydrochloric acid (10% HCl) prior to analysis. Plant P and soil total P contents were determined using the NaHCO_3_ alkali digestion method and by molybdenum antimony colorimetry (Cao et al. 2013). These determinations were carried out in the Key Laboratory of Mountain Surface Processes and Ecological Regulation at the Institute of Mountain Hazards and Environment, Chinese Academy of Sciences, China.

### Growing season climates data

Monthly meteorological datasets with spatial resolutions of 0.5° from 2005 to 2013 were derived from the China Meteorological Data Sharing Service System (http://cdc.nmic.cn). These meteorological gridded datasets were generated by thin plate spline (TPS) method using ANUSPLIN software (ERSI, Redlands, CA), and the data sources include monthly mean temperature and monthly precipitation data from more than 2400 well‐distributed climate stations across China, as well as digital elevation model (DEM) data. The average growing season (from May to September) temperature and growing season precipitation from 2005 to 2013 matched with nine sites' locations were extracted from these meteorological raster surfaces in ArcGIS 10.0 (ERSI) for further analyses.

### Data analysis

The Pielou evenness index, Shannon–Wiener diversity index, and Simpson dominance index were used to represent species diversity and were calculated using the methods presented by Schooler et al. (2006) and Wu et al. (2012).

The storage of C, N, and P in aboveground or belowground biomass was a product of the component biomass (kg m^−2^) and its respective mean concentration (g kg^−1^). The SOC pool at a depth of *i* (SOCP_*i*_, g m^−2^) was calculated as follows:

$${\text{SOCP}}_{i}=D_{i}\times B_{i}\times O_{i}\times 10$$

where *D*_*i*_ is the soil depth (cm), *B*_*i*_ is the soil bulk density (g cm^−3^), *O*_*i*_ is the SOC concentration at a depth of *i*, and 10 is the unit transfer coefficient.

SOC storage over *n* soil layers was calculated as follows:

$$\text{SOC storage}=\sum_{i\;=\;1}^{n}D_{i}\times B_{i}\times O_{i}\times 10$$

The storage of soil N and P was also computed using a similar formula.

A paired difference *t*‐test was used to test the potential effect of grazing exclusion on each ecosystem C, N, and P storage indicators. Analysis of covariance (ANCOVA) by the general linear model (GLM) was employed to evaluate the effects of grazing exclusion treatment and climatic factors on the C, N, and P storage parameters in Tibet. In the ANCOVA, the fixed factor was alpine grassland grazing treatments (FG and GE), while the covariates were growing season temperature and growing season precipitation. Homogeneity of variances and normal distribution of residuals were verified by examining plots of the distribution of residuals and of the residuals against fitted values to fulfill statistical assumptions of ANCOVA. The two covariates growing season temperature and growing season precipitation that were used to fit the linear ANCOVA models were not highly interacted with the fixed factor (*P *>* *0.05). The statistical analyses were performed using IBM SPSS Statistics 19 software (SPSS/IBM, Chicago, IL).

Redundancy analysis (RDA) using Canoco 4.5 and Canodraw for Windows (Microcomputer Power, Ithaca, New York, USA) was applied to examine the relationships between each ecosystem C, N, P storage indicators and the plant and soil environmental factors, including vegetation cover, vegetation height, the Pielou evenness index, the Shannon–Wiener diversity index, the Simpson dominance index, soil pH, bulk density, sand content (2–0.05 mm), silt content (0.05–0.002 mm), and clay content (<0.002 mm). RDA is a linear canonical ordination technique, that is, designed to detect the pattern of variation in response variables that can be associated with potential explanatory variables (Rabbi et al. 2014). According to the detrended correspondence analysis (DCA) results for the C, N, and P storage parameters in the ecosystem, the length of the gradient represented by axis 1 was 0.75. This finding indicated that the linear method works better than the unimodal method (Ter Braak and Smilauer 2002; Leps and Smilauer 2003). Thus, RDA was carried out for the relationships between C, N, and P storage indicators and the plant and soil factors in this study. The association between the storage of C, N, and P and the explanatory variables that had *P *<* *0.05 were considered statistically significant.

## Results

### Effect of grazing exclusion on ecosystem C storage

**Figure 1:**
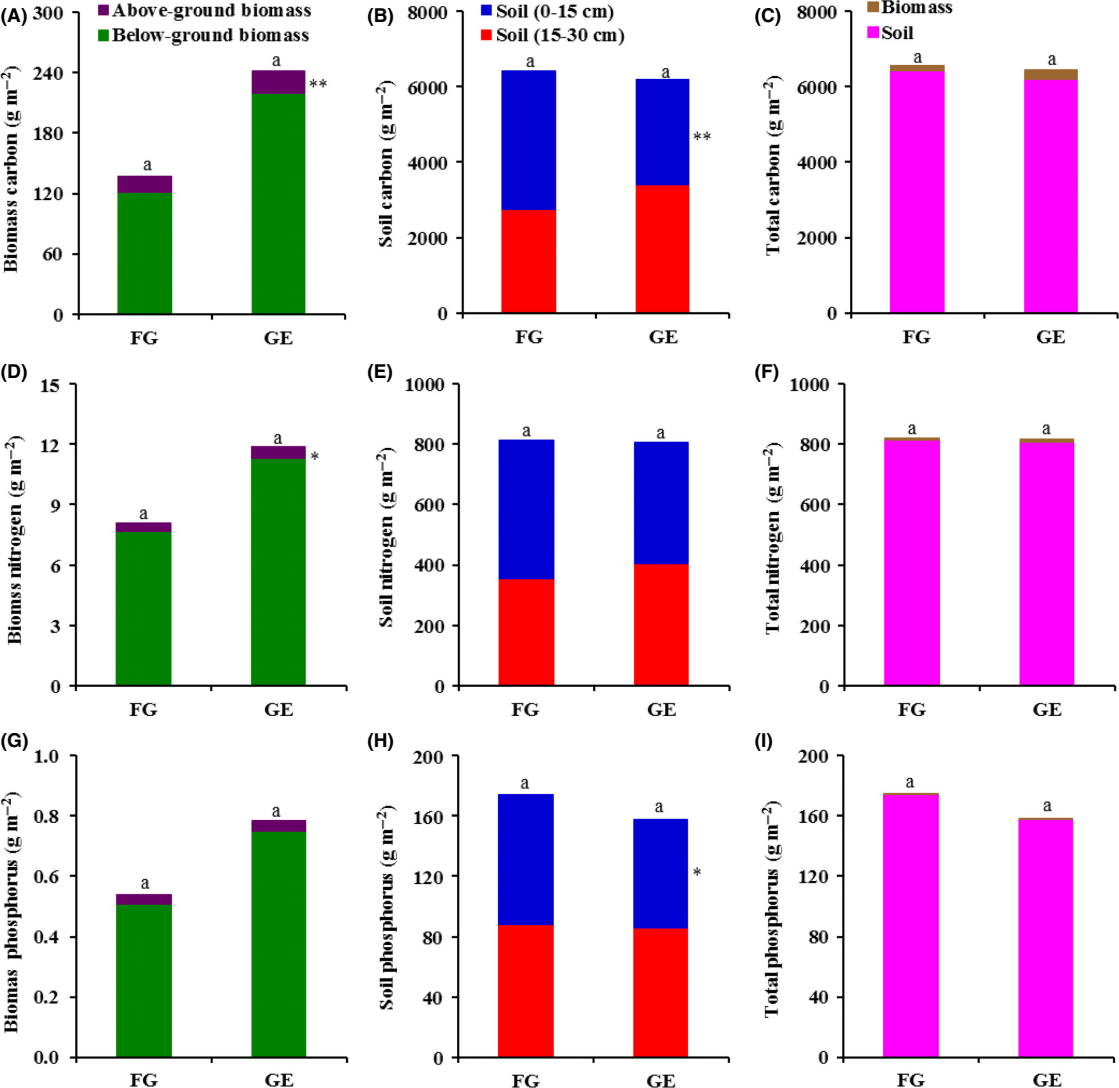
Effect of grazing exclusion (GE) and free grazing (FG) on C storage (A–C), N storage (D–F), and P storage (G–I) and their fractions in alpine grassland ecosystems. Note: Significant differences in C, N or P pools from different sources between GE and FG treatments are indicated by symbols next to the bars: **P < 0.01, *P < 0.05, no symbol, no significant difference. The same letters above the bars indicate insignificant differences in the C, N, or P storage between GE and FG grasslands.

The C stored in the aboveground biomass (AGBC) and the belowground biomass (BGBC) of alpine grasslands (alpine meadow + alpine steppe + alpine desert steppe) increased by 5.7 g m^−2^ (*P *<* *0.01) and 98.7 g m^−2^ (*P *=* *0.120) due to grazing exclusion treatment, respectively (Fig. 1A). Grazing exclusion also resulted in a significant decrease of 23.5% in the soil at a depth of 0–15 cm (*P *<* *0.01). However, the C stored in soil at a depth of 15–30 cm did not differ significantly between the GE and FG plots (Fig. 1B). In total, changes in both total biomass C (TBC: AGBC + BGBC) and total soil C (TSC: 0–30 cm) storage were not significant between GE and FG grasslands. The storage of total ecosystem C (TBC + TSC) in alpine grasslands were 6561.4 g m^−2^ for FG plots and 6452.5 g m^−2^ for GE plots, respectively; the difference between the two types of plots was also not significant (Fig. 1C). The total biomass C (TBC) storage accounted for a very small amount, which was 2.1% in FG plots and 3.7% in GE plots. Results from ANCOVA demonstrated that grazing exclusion only had a significant effect on AGBC, but had no effect on amount of total ecosystem C storage in alpine grasslands (Table 2). For the two climatic factors, growing season temperature had a significant negative effect on soil C pool at a depth of 0–15 cm, whereas growing season precipitation had a significant positive effect on all C storage fractions (Table 2).

**Table 2:** Results from analysis of covariance (ANCOVA) by the general linear model (GLM) showing *F* values and *P* values of ecosystem C, N, and P storage, which the fixed factor was grazing treatments (free grazing and grazing exclusion) and the covariates were growing season temperature and growing season precipitation. *P*‐values below 0.05 are in bold

C, N, P pools	Grazing treatment		Growing season temperature		Growing season precipitation	
	*F* value	*P* value	*F* value	*P* value	*F* value	*P* value
C pools						
AGBC	**5.1**	**0.029**	<0.1	0.979	**18.7**	**<0.001**
BGBC	3.0	0.092	0.1	0.811	**13.2**	**0.001**
TBC	3.3	0.075	0.1	0.810	**14.7**	**<0.001**
SC1	3.7	0.060	**4.8**	**0.034**	**6.0**	**0.018**
SC2	0.7	0.410	0.2	0.635	**4.4**	**0.040**
TSC	<0.1	0.845	0.3	0.579	**6.4**	**0.014**
TC	<0.1	0.921	0.3	0.589	**7.4**	**0.009**
N pools						
AGBN	1.8	0.190	0.1	0.798	**11.9**	**0.001**
BGBN	1.1	0.294	<0.1	0.987	**11.4**	**0.001**
TBN	1.2	0.278	<0.1	0.981	**12.1**	**0.001**
SN1	**9.6**	**0.003**	3.2	0.078	**8.0**	**0.007**
SN2	0.8	0.372	1.4	0.244	**7.2**	**0.010**
TSN	0.7	0.393	<0.1	0.970	**10.9**	**0.002**
TN	0.7	0.429	<0.1	0.971	**11.9**	**0.001**
P pools						
AGBP	0.2	0.666	0.2	0.634	**7.6**	**0.008**
BGBP	1.2	0.273	<0.1	0.885	**12.5**	**0.001**
TBP	1.3	0.267	<0.1	0.872	**13.2**	**0.001**
SP1	**5.0**	**0.030**	**24.5**	**<0.001**	**15.2**	**<0.001**
SP2	0.1	0.788	**14.7**	**<0.001**	**6.2**	**0.016**
TSP	1.6	0.215	**24.4**	**<0.001**	**12.6**	**0.001**
TP	1.5	0.221	**24.5**	**<0.001**	**12.2**	**0.001**

AGBC, C stored in aboveground biomass; AGBN, N stored in aboveground biomass; AGBP, P stored in aboveground biomass; BGBC, C stored in belowground biomass; BGBN, N stored in belowground biomass; BGBP, P stored in belowground biomass; TBC, C stored in total biomass; TBN, N stored in total biomass; TBP, P stored in total biomass; SC1, C stored in soil at 0–15 cm depth; SN1, N stored in soil at 0–15 cm depth; SP1, P stored in soil at 0–15 cm depth; SC2, C stored in soil at 15–30 cm depth; SN2, N stored in soil at 15–30 cm depth; SP2, P stored in soil at 15–30 cm depth; TSC, C stored in soil at 0–30 cm depth; TSN, N stored in soil at 0–30 cm depth; TSP, P stored in soil at 0–30 cm depth; TC, total C stored in ecosystem; TN, total N stored in ecosystem; TP, total P stored in ecosystem.

The effects of grazing exclusion on C storage were different among three different alpine grassland types (although note that the desert steppe is represented by a single site). Grazing exclusion in alpine meadow regions resulted in a significant reduction of 1486.1 g m^−2^ (*P *<* *0.05) in the soil C pool at a depth of 0–15 cm. However, grazing exclusion did not significantly affect other C pool fractions in the ecosystem (Table 3). In alpine steppe regions, grazing exclusion significantly increased the C stored in biomass; the C stored in the soil and the total ecosystem was not impacted. In alpine desert steppe regions, only AGBC was significantly greater in GE plots compared to FG plots (Table 3).

**Table 3:** Statistical comparison of overall mean values of ecosystem C, N, and P storage and their fractions between free grazing (FG) and grazing exclusion (GE) alpine grassland using paired difference *t*‐test (*α *= 0.05). ΔG means the values of GE plots minus the values of FG plots

C, N, P pools (g m^−2^)	Alpine meadow			Alpine steppe			Alpine desert steppe		
	FG	GE	ΔG	FG	GE	ΔG	FG	GE	ΔG
C pools									
AGBC	23.8	27.5	3.8	10.7	18.2	7.5**	6.7	12.5	5.9*
BGBC	196.6	395.8	199.2	52.9	74.2	21.3*	93.5	99.4	5.9
TBC	220.3	423.3	203.0	63.6	92.4	28.8*	100.1	111.9	11.8
SC1	5039.6	3552.5	−1487.1*	2424.5	2278.2	−146.3	3228.0	1995.3	−1232.8
SC2	3469.5	4830.0	1360.6	2162.3	2208.4	46.1	2205.3	2425.3	220.1
TSC	8509.1	8382.6	−126.5	4586.8	4486.7	−100.2	5433.3	4420.6	−1012.7
TC	8729.4	8805.9	76.5	4650.4	4579.0	−71.4	5533.4	4532.5	‐1000.9
N pools									
AGBN	0.7	0.7	0.1	0.3	0.5	0.2**	0.2	0.3	0.2*
BGBN	12.7	21.0	8.3	3.2	3.6	0.4	5.3	3.9	−1.4
TBN	13.3	21.7	8.3	3.5	4.0	0.5	5.5	4.2	−1.3
SN1	595.4	416.9	−178.5**	330.7	416.9	86.3	435.8	273.7	−162.1
SN2	433.9	533.9	100.0	283.0	289.2	6.2	304.3	349.8	45.6
TSN	1029.3	950.9	−78.5	613.7	563.9	−49.8	740.1	623.6	−116.5
TN	1042.6	972.5	−70.1	617.2	567.9	−49.3	745.5	627.7	−117.8
P pools									
AGBP	0.1	0.1	0.0	0.02	0.03	0.01*	0.01	0.01	0.0
BGBP	0.9	1.4	0.5	0.2	0.3	0.1	0.3	0.2	−0.1
TBP	1.0	1.5	0.5	0.2	0.3	0.1	0.3	0.2	−0.1
SP1	85.6	57.2	−28.4**	84.7	88.1	3.4	93.7	70.6	−23.1
SP2	86.5	74.6	−12.0	89.8	96.1	6.3	85.1	91.1	6.0
TSP	172.1	131.8	−40.4*	174.4	183.2	8.8	178.8	161.7	−17.1
TP	173.1	133.3	−39.9	174.7	183.5	8.8	179.0	161.9	−17.1

Abbreviations for ecosystem C, N, and P storage and their fractions as in Table 2.

*P < 0.05, **P < 0.01.

### Effect of grazing exclusion on ecosystem N and P storage

The N stored in the aboveground biomass (AGBN) significantly increased due to grazing exclusion (*P *<* *0.05), although it accounted for a negligible amount (<0.1% of the total) of total N storage in the ecosystem (Fig. 1D). Nevertheless, the N stored in belowground biomass (BGBN), total biomass (TBN), soil N at both 0–15 and 15–30 cm, and the total soil N (TSN) did not differ significantly between GE and FG grasslands (Fig. 1D, E). Compared to the FG grasslands, the soil P pools at a depth of 0–15 cm was 15.9% lower in the GE grasslands (*P *<* *0.05). The P stored in biomass and the soil at a depth of 15–30 cm was not significantly altered due to grazing exclusion treatment (Fig. 1G, H). There were no significant differences in the response of total N storage or total P storage in the alpine grassland ecosystem following GE treatment (Fig. 1F, I). Statistical analyses from ANCOVA showed that ecosystem N and P storage indictors were not significantly impacted by grazing exclusion, except for soil N and P pools at a depth of 0–15 cm, which were significantly different between the FG and GE plots (Table 2). For the climatic factors, growing season temperature had a significant negative effect on soil and total P storage, whereas growing season precipitation had a significant positive effect on all ecosystem N storage fractions and biomass P storage, but had a significant negative effect on soil P storage fractions (Table 2).

Similar to C storage, grazing exclusion also has the different effects on N and P storage among different alpine grassland types. In alpine meadow regions grazing exclusion significantly decreased the soil N and P pools at a depth of 0–15 cm and the total soil P pool (TSP: 0–30 cm), but had no impacted other N and P storage indicators. In alpine steppe regions, the N and P stored in the aboveground biomass (AGBN and AGBP) significantly increased due to grazing exclusion treatment, the N and P stored in the belowground biomass and soil were not impacted. In alpine desert steppe regions, only the AGBN storage was significant greater in GE plots compared to FG plots (Table 3).

### Relationships between ecosystem C, N, P storage and plant and soil factors

**Figure 2:**
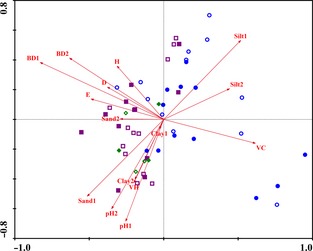
Redundancy analysis (RDA) of ecosystem C, N, and P storage and environmental variables (plant and soil environmental factors) from the three alpine grassland types with grazing exclusion (GE) or free grazing (FG) treatments. The blue cycle represents the alpine meadow, the purple square represents the alpine steppe, the green rhombus represents the alpine desert steppe, hollow shapes represents FG grassland, and solid shapes represent GE grasslands. VC: vegetation cover, VH: vegetation height, D: Simpson dominance index, H: Shannon–Wiener diversity index, E: Pielou evenness index, pH1: soil pH at a depth of 0–15 cm, pH2: soil pH at a depth of 15–30 cm, BD1: soil bulk density at a depth of 0–15 cm, BD2: soil bulk density at a depth of 15–30 cm, Sand1: soil sand content at a depth of 0–15 cm, Sand2: soil sand content at a depth of 15–30 cm, Silt1: soil silt content at a depth of 0–15 cm, Silt2: soil silt content at a depth of 15–30 cm, Clay1: soil clay content at a depth of 0–15 cm, Clay2: soil clay content at a depth of 15–30 cm.

Eigenvalues of the RDA indicated that 66.4% of the total variance within C, N, and P storage in alpine grassland ecosystems was explained by the first two axes (*F *=* *6.1, *P *=* *0.002). The first ordination axis explained 64.0% of the total data variance, whereas the second explains 2.4% (Fig. 2). Species–environment correlations for the first two canonical axes of the RDA were 0.99, indicating that the storage of ecosystem C, N, and P was strongly correlated with plant and soil environmental parameters. Vegetation cover and soil silt content at both 0–15 cm and 15–30 cm positively contributed to the formation of this first axis (*P *<* *0.05). Simpson dominance index, Pielou evenness index, and the soil bulk density at 0–15 cm and 15–30 cm, the soil sand content at 0–15 cm, and the soil PH at 15–30 cm negatively contributed to the formation of this first axis (*P *<* *0.05). The formation of a second canonical axis can be defined by the positive contributions of the soil silt content at 0–15 cm and the soil bulk density at 15–30 cm (*P *<* *0.05), and the negative contributions of vegetation height, the soil sand content at 0–15 cm, the soil clay content at 15–30 cm, and the soil pH at both 0–15 cm and 15–30 cm (*P *<* *0.05) (Fig. 2).

**Figure 3:**
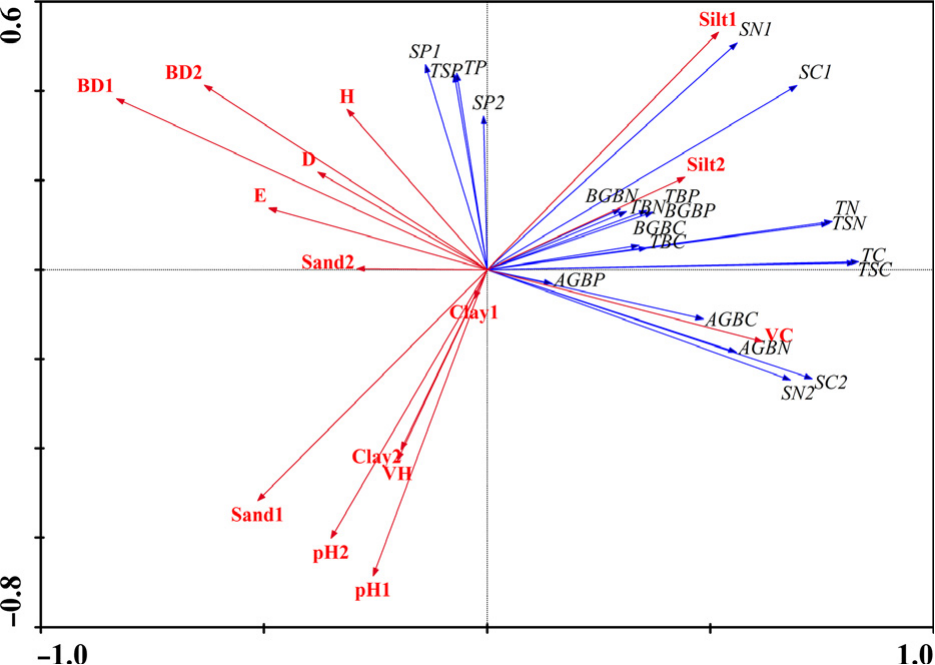
Redundancy analysis (RDA) ordination diagram for ecosystem C, N, and P storage in relation to plant and soil environmental factors in the alpine grassland of Tibet. Abbreviations for plant and soil environmental parameters are as in Fig. 2; the abbreviations for ecosystem C, N, and P storage and their fractions are as in Table 2.

The cosines of angles between ecosystem C, N, and P storage and environmental variables reflect their relationships. All C and N storage values in biomass and soil, as well as total ecosystem C and N storage values, were positively influenced along the first axis. The P stored in biomass was positively influenced along the first axis; the P stored in soil and the total ecosystems were positively influenced along the second canonical axis (Fig. 3). The shorter arrow observed for AGBP, BGBC, BGBN, BGBP, TBC, TBN, TBP, and SP storage at 15–30 cm indicates that these values were not strongly influenced by plant and soil environmental factors. The variable arrows for the plant and soil environmental parameters point in approximately the same direction as the ecosystem C, N, and P storage arrows, indicating a high positive correlation. In general, AGBC storage and AGBN storage were positively correlated with vegetation cover and negatively correlated with Simpson dominance index, Shannon–Wiener diversity index, and Pielou evenness index. The storage of soil C, N, and P at a depth of 0–15 cm was positively correlated with soil silt content and negatively correlated with soil sand content and soil pH. The storage of soil C and N at 15–30 cm was largely negatively influenced by soil bulk density.

## Discussion

Grazing exclusion was expected to lead to greater ecosystem C storage due to the removal of disturbance by grazing animals. Overall, our study showed lack of significant difference in total C storage between FG and GE plots, which indicated no impact on ecosystem C storage of alpine grasslands due to grazing exclusion in Tibet. This result agrees well with Nosetto et al. (2006), who found that grazing exclusion in a semiarid ecosystem in Argentina did not result in significant changes in the total ecosystem C storage after 15 years of grazing exclusion. However, our results were not consistent with previous studies conducted in the Inner Mongolia region of China; in these experiments, grazing exclusion resulted in significant increases in total C storage by 15–350% in the grassland ecosystem under different restoration periods (He et al. 2008; Li et al. 2012; Wu et al. 2014a;). Our findings were also not consistent with results from the three headwater resource regions in the Tibetan Plateau, in which the total C storage in an alpine meadow ecosystem increased by 26.8% after being fenced for 5 years (Fan et al. 2013). These inconsistent results may be caused by site differences in regional climate, environmental conditions, the duration of grazing exclusion, soil and vegetation types, and the degree of degradation (Li et al. 2012).

The C stored in the aboveground tissues is a time‐integrated expression of ecological and land‐use processes ranging from photosynthesis and nutrient cycling to disturbance and climate change; the C stored in roots is an important element in soil carbon sequestration (Johnston et al. 2004; Asner et al. 2012; Li et al. 2013). In our study, AGBC storage significant increased with GE treatment. An insignificant increase in BGBC storage was also observed (Fig. 1A). These results suggested that grazing exclusion in alpine grasslands had the potential to enhance the accumulation of C in biomass, especially in aboveground tissues. Similar findings were reported for alpine meadow regions east of the Tibetan plateau by Li et al. (2013); grazing exclusion directly increased biomass C in moderately degraded grassland. Fan et al. (2013) reported that C storage in aboveground tissue and roots increased by 30.9% and 63.2%, respectively, after being fenced for 5 years. An increase in the C stored in biomass is probably observed because biomass is not removed or destroyed by herbivores. For instance, grazing exclusion can increase the growth and development of annual and perennial grasses and dense rooting systems; this in turn supports vegetation C accumulation in alpine grasslands (Zhou et al. 2006; Wu et al. 2009).

Total C stored in the soil (0–30 cm) was not significantly altered by the exclusion of grazing in alpine grasslands. However, C storage in the soil surface layer (0–15 cm) significantly decreased by 862.9 g m^−2^ (Fig. 1B). This decrease was in agreement with two former studies from the Tibetan Plateau. The SOC stocks in the soil surface layer were reduced by 25% after 8 years of grazing exclusion in Tianzhu (Shi et al. 2013). The SOC stocks in soil layers at depth of 0–5 and 5–15 cm were reduced by 21% and 30%, respectively, after 7 years without grazing in Xinghai (Hafner et al. 2012). Bulk densities used in calculations of change in SOC storage are of critical importance because only a small change in bulk density can result in great change in stock of SOC (Smith et al. 2007). In alpine grasslands, we found that the average bulk densities and SOC concentrations of soil surface layer decreased 0.1 g cm^−3^ and 1.8 g kg^−1^, respectively, due to grazing exclusion; nevertheless, these reductions were not statistically significant. However, the SOC storage in the soil surface layer, which calculated from soil bulk densities and SOC concentrations, was significantly lower in GE plots than that in FG plots. Possible explanations for how grazing exclusion reduces C storage in the soil surface layer are (1) the absence of excreta inputs when the conventional grazing by yak, sheep and goats, and large wildlife herbivores are excluded; (2) aboveground litter accumulates on the soil surface in the absence of grazing sites resulting in carbon immobilization; therefore, C incorporation into soil is decreased due to the absence of physical breakdown; (3) less incorporation of root‐derived carbon into stable soil carbon pools because roots from grazed site have lower cell solubles, higher lignin:N ratios, and higher tannin content than plants from the ungrazed sites; and (4) increase in aboveground biomass drive greater competition for nutrients, further drive more soil organic matter mineralization and accompany with greater carbon emission (Smith et al. 2007; Semmartin et al. 2008; Hafner et al. 2012; Schrama et al. 2013; Shi et al. 2013; Chen et al. 2015).

Nevertheless, some studies reported that SOC storage in the soil surface layer increased significantly due to grazing exclusion treatment in alpine grasslands on the Tibetan Plateau (Wu et al. 2010; Fan et al. 2013; Li et al. 2013). All of the studies in which a positive effect on SOC storage were observed on the Tibetan Plateau were conducted in alpine meadow ecosystems and in one single experimental site. In the present study, the data are on a regional scale; we included three alpine grassland types and nine research sites. In fact, the effects of grazing exclusion on SOC of grassland ecosystems from different studies were shown to be contradictory all over the world; in various cases, they have demonstrated a positive effect, a negative effect, and a neutral effect probably because of the contributions of different grassland types, inconsistent years of grazing exclusion, soil heterogeneity, and different environmental conditions (Mekuria and Aynekulu 2013; Raiesi and Riahi 2014; Speed et al. 2014).

In alpine grassland regions, grazing exclusion treatment affected ecosystem N and P storage in a manner similar to C storage. Grazing exclusion had no significant influence on total ecosystem N storage and soil N storage. However, AGBN storage significantly increased by 27.3% in GE grasslands. Fan et al. (2013) found the N pools from both biomass and the soil increased after grazing exclusion in the three headwater resource regions. In contrast, Shi et al. (2013) reported the N stored in both biomass and the soil decreased in GE grasslands east of the Tibetan Plateau. That N stocks can either increase or decrease probably results from the balance between changes in belowground N allocation patterns (the root N retention hypothesis) and the ability of the soil to retain the extra N available after the exclusion of herbivores and the cessation of volatilization and leaching from urine and dung patches (the N loss hypothesis) (Piñeiro et al. 2006, 2009).

We found that the P stored in biomass and the total P stored in the ecosystem were not significant different between FG and GE plots. Soil P pools at a depth of 0–15 cm significantly decreased by 15.8% in GE grasslands (Fig. 1H). The reduction of P pools in soil surface layer due to grazing exclusion maybe contributed by the absence of inputs of animal excreta, which has long been recognized as an important pathway in the P cycle in grazed pasture, and higher soil P uptake by vegetation (Rowarth et al. 1985; Chaneton and Lavado 1996). Nevertheless, higher soil P content inside fenced areas was also reported on the Tibetan Plateau (Wu et al. 2009) and in grasslands of other regions (Pei et al. 2008; Wesche et al. 2010), for which the possible explanations were the trapping of fine material by the denser litter and the standing vegetation (Pei et al. 2008) and the reduction of the outflow of energy and nutrients from soil–plant system to consumers (Wu et al. 2009).

Results from ANCOVA demonstrated that precipitation during the growing season played an important role in controlling the C, N, and P storage of alpine grasslands in Tibet because growing season precipitation was found had significant effects on all C, N, and P storage (Table 2). There is increasing evidence to show that precipitation controls the spatial distribution of species richness and diversity, primary production, and carbon and water cycles of alpine grassland ecosystems in this region (Hu et al. 2010; Yang et al. 2010; Wu et al. 2012, 2013). The potential changes in precipitation are identified as important aspects of regional climate change, which can alter the distribution and dynamics of water availability and subsequently alter vegetation growth and soil biogeochemical processes at the ecosystem level (Hao et al. 2013; Nielsen and Ball 2015). The total amount of precipitation and altered precipitation patterns could play the most prominent role in grassland ecosystem C, N and P dynamics, especially for arid and semiarid ecosystems, through their influence on plant productivity (Heisler‐White et al. 2008; Robertson et al. 2009), soil carbon cycle processes (Hao et al. 2013; Manning et al. 2015), and soil N and P transformations (Ippolito et al. 2010; Cregger et al. 2014). In this case, the storage of C, N, and P of alpine grasslands was primarily driven by the growing season precipitation gradient, whereas grazing exclusion almost had no effect on ecosystem C, N, and P storage and their fractions (Table 2). Similar results were also reported by Wu et al. (2012, 2014b) in this region; therefore, the potential shift of growing season precipitation in Tibet should be considered when considering policies designed for the carbon management of alpine grasslands in the future.

The RDA, a multivariate technique, was used to identify variation in ecosystem C, N, and P storage and fractions in alpine grasslands across a range of vegetation characteristics and soil properties. The ecosystem C, N, and P storage among different alpine grassland types and different grazing regimes had no clear distribution associated with plant and soil environmental parameters (Fig. 2). However, the C, N, and P stored in aboveground biomass were found to be positively correlated with vegetation cover and negatively correlated with Simpson dominance index, Shannon–Wiener diversity index, and Pielou evenness index (Fig. 3). This is because the storage of AGBC, AGBN, and AGBP depends on aboveground biomass; several studies have demonstrated that biomass and vegetation cover simultaneously increase in fenced grasslands due to the absence of disturbances from herbivorous livestock (Jeddi and Chaieb 2010). The biodiversity index decreases due to the loss of lower competitive plant species in fenced alpine grassland ecosystems (Wu et al. 2009; Dorji et al. 2010; Xiong et al. 2014). In addition, the storage of soil C, N, and P at a depth of 0–15 cm was positively correlated with soil silt content and negatively correlated with soil sand content (Fig. 3). The amount of soil organic matter and soil nutrients are associated with silt and clay due to their greater capacity for holding water and nutrients compared to sand (Carter 2002; Plante et al. 2006). Soil clay content is very low in alpine grassland, so it plays a negligible role in soil C, N, and P storage.

## Conclusions

In general, short‐term grazing exclusion (6–8 years) by fencing had no effect on total C, N, and P storage in alpine grassland ecosystems. In addition, grazing exclusion did not affect the C, N, and P stored in both total biomass and soil (0–30 cm) fractions. The ANCOVA also demonstrated that grazing exclusion almost had no significant effect on ecosystem C, N, and P storage and their fractions in alpine grasslands, but growing season precipitation had a significant effect on all ecosystem C, N, and P storage indicators, which indicate, that precipitation is a key factor controlling C, N, and P storage and distribution in this alpine grasslands. Of total ecosystem C, N, and P storage, the percentage of C, N, and P stored in biomass are very small (<4%) and soil C, N, and P storage are the primary components of total ecosystem C, N, and P storage. The C, N, and P stored in aboveground biomass increased due to grazing exclusion; this was especially obvious in alpine steppe and alpine desert steppe regions. However, these increases could not compensate for the considerable loss of C, N, and P pools from the soil surface layer, which led to the total C, N, and P storage in alpine grassland ecosystem decreased by 108.9, 66.1, and 15.7 g m^−2^ following grazing exclusion, respectively; even through these decreases were not statistically significant. Thus, we predict that grazing exclusion could potentially significantly decrease total C, N, and P storage in alpine grassland ecosystems if continuous extending grazing exclusion continues for years. However, to support this hypothesis, it would be necessary to investigate the responses of alpine grassland C, N, and P storage to long‐term grazing exclusion for longer periods of time.

## Conflict of Interest

The authors have declared that no competing interests exist.
